# Resistance Training in Patients After Metabolic and Bariatric Surgery: Protocol for a Systematic Review

**DOI:** 10.2196/49513

**Published:** 2023-11-23

**Authors:** Whitney Elks, Adam Rooks, Spencer Schulte, Kavita Batra, Jocelyn Burke, Varun Jain

**Affiliations:** 1 Department of Medical Education Kirk Kerkorian School of Medicine at University of Nevada, Las Vegas Las Vegas, NV United States; 2 Department of Surgery University of Kentucky College of Medicine Lexington, KY United States; 3

**Keywords:** bariatric surgery, bariatric, body composition, exercise, meta-analyses, meta-analysis, muscle, muscular, physical activity, physiotherapy, post-metabolic, resistance training, resistance, review methodology, review methods, systematic, weight lifting, weight

## Abstract

**Background:**

Resistance training has been consistently shown to have multiple health benefits, especially for patients who have undergone bariatric surgery. Patients who have undergone bariatric surgery are recommended to participate in resistance exercise; however, protocols and guidelines for resistance training remain poorly implemented.

**Objective:**

This is a protocol for a systematic review and possibly a meta-analysis that will synthesize evidence of the effects of resistance exercise on changes in body composition, muscular strength, overall weight loss or maintenance of weight loss, and quality of life in patients after metabolic and bariatric surgery (MBS). The findings of this study may provide practice recommendations for resistance training among patients who have undergone MBS.

**Methods:**

We registered this systematic review on PROSPERO (CRD42023464928) on September 18, 2023. A systematic search of electronic databases (Embase, PubMed, Scopus, Web of Science, and CINAHL) was conducted on studies published from January 1, 1991, to May 15, 2023, to identify English-language human studies on adult patients who have undergone MBS that include a resistance training intervention and describe outcome measurements of body composition or strength. Screening will be performed using PRISMA (Preferred Reporting Items for Systematic Reviews and Meta-Analyses) guidelines, and relevant data elements will be extracted.

**Results:**

Searches and screenings commenced in May 2023. Data extraction and analyses will be completed by the end of December 2023, after which findings will be synthesized and reported by the end of March 2024.

**Conclusions:**

This systematic review will summarize the evidence regarding resistance training in patients after MBS. The findings from this systematic review and possible meta-analysis may provide practice recommendations for resistance training protocols in this patient population and identify characteristics of protocols with the best adherence and outcomes. With these results, we anticipate that we will gain a deeper understanding of the role of resistance training after MBS.

**Trial Registration:**

PROSPERO CRD42023464928; https://www.crd.york.ac.uk/prospero/display_record.php?RecordID=464928

**International Registered Report Identifier (IRRID):**

PRR1-10.2196/49513

## Introduction

Obesity negatively affects nearly every organ system. Since the National Institutes of Health published a statement on gastrointestinal surgery for severe obesity in 1991, metabolic and bariatric surgery (MBS) has been extensively studied over the last 30 years. Surgical procedures as well as perioperative protocols continue to evolve. The benefits and favorable outcomes of MBS have been consistently proven to be durable for up to 20 years after surgery. These outcomes include greater than 60% excess body weight loss (%EBWL); superiority over diet, exercise, and other lifestyle interventions in attaining significant and durable weight loss; improvement in obesity-related comorbid conditions such as glycemic control in type 2 diabetes mellitus [1], obesity-associated cancers [2], and nonalcoholic steatohepatitis [3]; and improvement in quality of life and overall mortality [2,4]. The evidence has been so robust that the American Society for Metabolic and Bariatric Surgery and the International Federation for the Surgery of Obesity and Metabolic Disorders have convened to produce a joint statement on the current available scientific information on MBS in 2022, which has expanded the indications for MBS to include individuals with a BMI ≥35 kg/m^2^, regardless of the presence, absence, or severity of comorbidities; individuals with metabolic disease and a BMI of 30-34.9 kg/m^2^; individuals in the Asian population with a BMI ≥27.5 kg/m^2^; and appropriately selected children and adolescents [4].

To augment the health benefits of MBS, patients who have undergone bariatric surgery are recommended to participate in regular physical activity [5], although the recommended guidelines remain somewhat nonspecific. The American College of Sports Medicine recommends the same amount of physical activity for both individuals with obesity and patients who have undergone bariatric surgery [6]. Patients who have undergone MBS may require different recommendations than those who have not undergone surgery, as patients after MBS consume what is considered a very low-calorie diet and experience large and rapid weight loss. The rapid weight loss is not only fat mass, but it has been shown to also be fat-free mass, including muscle mass, which is associated with a decrease in muscle strength [7,8].

Resistance training is defined as a form of physical activity that is designed to improve muscular fitness by exercising a muscle or a muscle group against any external resistance [6]. This can include modalities such as machines, free weights, bands, and body weight movements. Resistance training should be implemented to mitigate sarcopenia or skeletal muscle loss, which has in turn been shown to improve strength in patients who have undergone MBS [9-11]. Resistance exercise has been proven to have many benefits. It may assist in the prevention and management of type 2 diabetes by decreasing visceral fat and improving insulin sensitivity [12,13]. It improves physical performance, movement control, walking speed, functional independence, cognitive capabilities, and self-esteem [13,14]. It has also been shown to improve balance and reduce the risk of falls in older adults, which may be a major cause of morbidity and death [15]. Therefore, muscle mass and strength are arguably the most crucial elements of body composition, given their contribution to independence and longevity.

Studies have shown that maintenance of (and in some cases gains in) muscle strength occurred in the context of massive lean body mass loss in patients who have undergone MBS [5,9,10]. The implementation of effective resistance training programs specifically designed for patients who have undergone MBS could help this patient population with favorable body recomposition by increasing fat loss while retaining and even building lean mass, bone mass, and muscular strength. Efforts toward creating specific recommendations for resistance training protocols in patients after MBS have been made based on a systematic review by Morales-Marroquin et al [16]. However, this review was small and based on 9 randomized controlled trials. The effects of resistance exercise in patients after MBS have not yet been assessed through meta-analysis.

With the new 2022 statement on the expansion of patients who should be considered for MBS in mind, the population of patients who have undergone MBS will likely continue to increase, necessitating clearer recommendations and practice guidelines for resistance training in this population.

This study aims to be the largest and most comprehensive systematic review of resistance training protocols and their effects on patients who have undergone MBS with regard to body composition, muscular strength, and relevant health outcomes. More importantly, it also aims to review which protocols have superior adherence and compliance after the implementation of resistance training. Lastly, we hope to offer practice recommendations for resistance training protocols that can be applied in this patient population with the ultimate goal of long-term healthy weight loss and weight maintenance.

## Methods

### Ethical Considerations

As this study does not have the direct involvement of human participants, an institutional ethical review will not be warranted. However, to adhere to a strict methodology, the protocol of this systematic review and meta-analysis has been registered on PROSPERO (CRD42023464928) [17]. PROSPERO is an international database of prospectively registered systematic reviews, which provides a unique permanent registration number to the protocol that prevents duplication, thereby reducing reporting bias.

### Review Question

Our review question is as follows: “What are the effects of resistance training on patients after MBS? Additionally, which resistance training intervention demonstrated the most improvement regarding body composition and relevant health outcomes, and which intervention had the best adherence?”

### Inclusion and Exclusion Criteria

The PECOS (Population, Experiment, Comparator, Outcome, Study Design) framework was used to formulate eligibility criteria for this systematic review and meta-analysis [18]. Case series, case-control studies, retrospective and prospective cohorts, and randomized and nonrandomized clinical trials will be eligible for inclusion. Studies published in other languages, case studies, editorials, posters, abstract-only studies, conference proceedings, animal studies, and reviews will be excluded. Studies that do not include resistance training as a component of the exercise intervention or those without quantitative data on outcomes of interest will also be excluded. We will use the PRISMA (Preferred Reporting Items for Systematic Reviews and Meta-Analyses) checklist.

**Figure 1:**
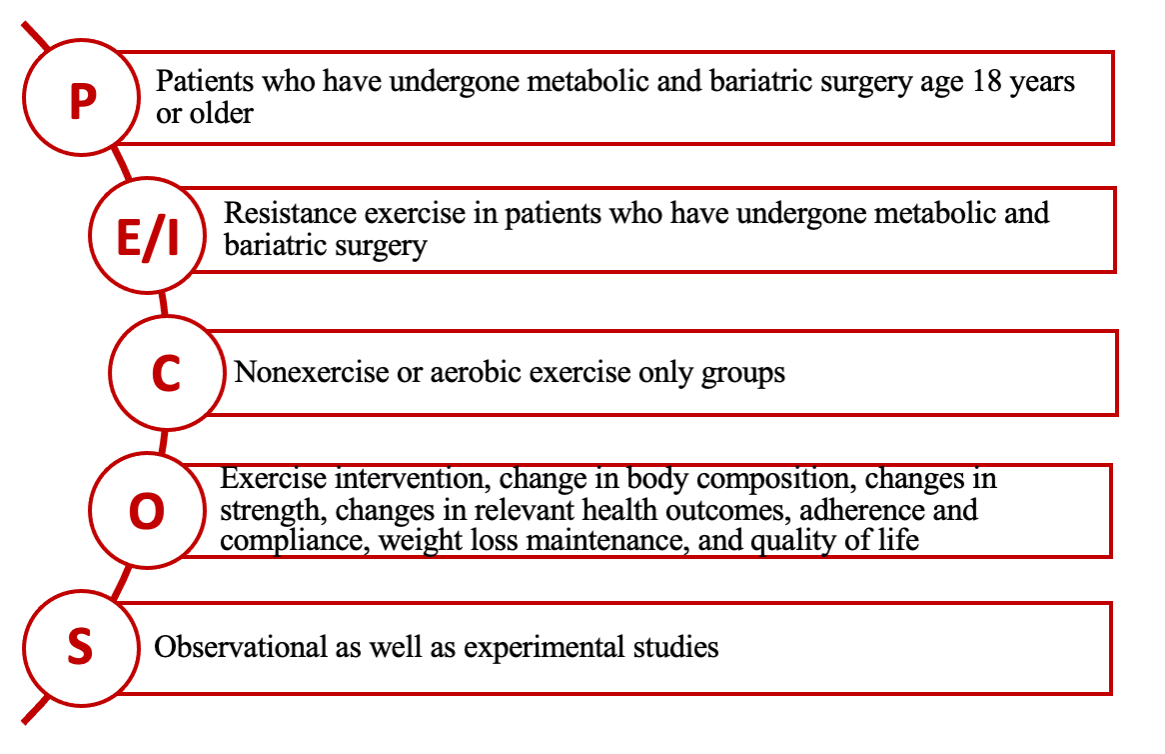
PECOS (Population, Experiment, Comparator, Outcome, Study Design) framework of the study.

Study eligibility criteria in the form of PECOS criteria are as follows (Figure 1): we will include studies with adult patients aged 18 years or older; individuals who have undergone MBS, including the following procedures—Roux-en-Y gastric bypass, sleeve gastrectomy, biliopancreatic diversion with duodenal switch, single anastomosis duodenal-ileal bypass with sleeve gastrectomy, gastric band, or revisional bariatric surgery; and those who have undergone a resistance exercise intervention; studies that include outcome measurements of body composition or strength; and articles that were published in English and were performed on humans. We will exclude studies that were not performed on adult human participants, are not in English, included patients who have not undergone MBS, did not include body composition or strength components as outcome measures, and had no resistance exercise component in the intervention.

### Informational Sources and Search Strategy

A systematic search of bibliographical electronic databases was conducted on studies published from January 1, 1991, to May 15, 2023, to identify English-language human studies. A comprehensive search and article retrieval strategy was planned by the study’s investigators, guided by a library subject matter expert, to find potentially relevant articles in the following databases: Embase, PubMed, Scopus, Web of Science, and CINAHL. In accordance with the search development and optimization method proposed by Bramer et al [19], the search strategy was developed in Embase and then translated into the syntax of each of the other databases. The search strategy was optimized for each database using a thesaurus, free-text search terms, as well as Medical Subject Headings terms. Informed by the PECOS framework (Figure 1), search strings for each database were developed iteratively and revised as new search terms were discovered. The search strategy will be peer-reviewed by a second research librarian using the Peer Review of Electronic Search Strategies (PRESS) 2015 Evidence-Based Checklist [20].

Multimedia Appendix 1 includes the full search strategy in detail. Keyword criteria included combinations of the following words and phrases: bariatric surgery, gastric bypass, sleeve gastrectomy, gastric band, biliopancreatic diversion with duodenal switch*,* resistance training, and weight lifting. We will include studies on humans and in English from all geographical locations.

### Screening

All records or articles will be imported into an intelligent, systematic review tool called Rayyan (Rayyan Systems Inc) for screening. After the deduplication, all records will be assessed by 2 independent reviewers to check for inclusion and exclusion criteria (Figure 1). Titles, abstracts, and full texts of articles will be screened in a systematic and sequential manner. All reasons for exclusion will be documented at each step of screening. A PRISMA flow diagram will be used for describing the study selection process [21].

### Data Extraction and Main Data Elements

A total of 2 reviewers will independently extract the relevant data elements from the eligible full texts of the articles and record these variables in a standardized code book. A double extraction method will be used to ensure accuracy and completeness. After data extraction, disparities will be resolved by consensus and discussion with the third reviewer, or a “tiebreaker.” Attempts to contact the corresponding authors of the included articles will be made if more information about the individual study’s data is needed. The following data elements will be extracted: (1) study title; (2) study author; (3) year; (4) evidence level; (5) quality score; (6) study design; (7) sample size; (8) sex (menopausal status, if in female individuals); (9) age; (10) geographic location (country); (11) operation (Roux-en-Y gastric bypass, sleeve gastrectomy, gastric band, revision bariatric surgery, biliopancreatic diversion with duodenal switch, or single anastomosis duodenal-ileal bypass with sleeve); (12) exercise intervention (type of exercise training [resistance, resistance + aerobic, aerobic only, or none]), postoperative initiation of intervention, frequency (days per week and minutes per session), intensity (sets and repetitions), and the duration of program (weeks or months); (13) exercise setting (supervised, nonsupervised, individual, group, and combined); (14) diet (protein intake); (15) key findings; (16) changes in body composition (%EBWL, total body weight loss [%TBWL], fat mass, fat-free mass, muscle mass, strength, and bone density); (16) how changes in body composition were measured; (17) how strength was measured; (18) changes in relevant health outcomes (heart disease, diabetes mellitus, obstructive sleep apnea, and osteoarthritis or degenerative joint disease); (19) measure of nonexercise physical activity; (20) adherence or compliance; (21) weight loss maintenance; (22) quality of life; (23) self-esteem; (24) habitual physical activity; and (25) length of follow-up.

The primary outcomes of interest in this study are changes in body composition (%EBWL, %TBWL, fat mass, fat-free mass, muscle mass, strength, and bone density). The secondary outcomes of interest include (1) relevant health outcomes (heart disease, diabetes mellitus, obstructive sleep apnea, and osteoarthritis or degenerative joint disease), (2) adherence and compliance, and (3) quality of life and self-esteem.

### Quality or Risk of Bias Assessment

**Figure 2:**
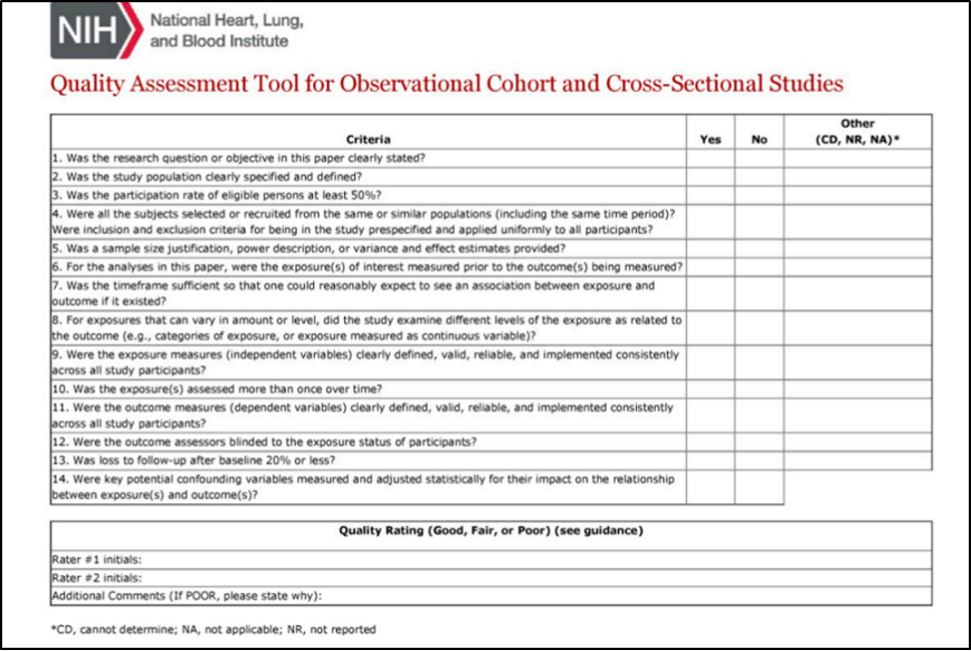
National Heart, Lung, and Blood Institute health quality assessment tool for observational, cohort, and cross-sectional studies.

For the quality assessments of the included studies, the National Heart, Lung, and Blood Institute quality assessment tool will be used. A total of 2 reviewers will assess the quality of the full texts and will perform scoring independently. The kappa statistics will be calculated to measure the interrater agreement. The National Heart, Lung, and Blood Institute tool has 14 items in the checklist (Figure 2) to evaluate all essential components of original research studies. Quality will be rated as poor (0-4 out of 14 questions), fair (5-10 out of 14 questions), or good (11-14 out of 14 questions) as guided by the tool.

### Statistical Plan

The first results of all the finally included studies will be described succinctly in the form of a summary table. The pooled estimates with a 95% CI will be generated using the Comprehensive Meta-Analysis software (version 3.0; Biostat). A random-effects model will be used to calculate pooled estimates, as this is a more robust estimate regardless of heterogeneity [22]. Cochran Q and *I*^2^ statistics will be used as indicators of heterogeneity. If enough studies are available in subgroups, then further analyses based on moderator variables, such as sociodemographic variables, country, and type of intervention, will also be conducted. Sensitivity analysis will be conducted to identify studies that may severely affect the pooled prevalence. A funnel plot and Egger linear regression test will be used to assess publication bias [23]. The significant level will be set as 2-sided and *P*<.05. Forest plots will be used to present the data.

### Data Analysis and Presentation

We will present the extracted data in tabular form and as a systematic summary as required. A data extraction form will be developed before article analysis. The table will report the extracted variables as listed above in the *Data Extraction and Main Data Elements* section. If the findings are poolable, quantitative analysis will be conducted.

Our meta-analysis plan is as follows: the number of studies with positive, null, or negative findings will be presented. Effects of resistance exercise on changes in body weight, fat mass, lean body mass, muscle strength, quality of life, changes in health outcomes, and adherence will be examined using random effects models. The Comprehensive Meta-Analysis software will be used to analyze the data. The mean (SD) of the absolute change in the intervention and control groups will be reported. Pooled-effect estimates (effect size) will be expressed as the weighted mean difference (MD) between exercise and control groups for the outcomes listed above and as the weighted standardized MD for changes in the outcomes. *P*<.05 will be considered statistically significant. Heterogeneity will be assessed using *I*^2^, with values interpreted as low at 25%, moderate at 50%, and high at 75%. The τ^2^ and test of homogeneity will also be reported. Prediction intervals (PIs) will be calculated using the formula 95% PI = MD ± 2 τ^2^. To identify sources of heterogeneity, sensitivity analyses with the one-study-removed procedure will be performed. Publication bias will be assessed with a visual inspection of the funnel plot and Egger regression test.

The reason a meta-analysis is a possibility and not definite is because we do not yet know if the group of studies will be sufficiently homogenous in terms of subjects involved, type of intervention (eg, resistance training), outcome, and the type of effect measure reported in each study to provide a meaningful summary. In other words, if studies are too heterogeneous to be comparable, the true effects in meta-analytical evidence may be obscured. It is possible to produce a narrative synthesis (systematic review) of the entire pool of studies and perform quantitative analysis (meta-analysis) only on the randomized controlled trials.

## Results

The search for this systematic review commenced in May 2023. Data extraction and analyses will be completed by the end of December 2023, after which findings will be synthesized and reported by the end of March 2024. The plan for dissemination, however, is to publish the review’s findings in a peer-reviewed journal and present findings at high-level conferences to share knowledge to help establish specific resistance exercise protocols in patients after MBS. A timeline is provided in Table 1.

**Table 1:** Timeline table for review.

Tasks	May 2023	Jun 2023	July 2023	Aug 2023	Sept 2023	Oct 2023	Nov 2023	Dec 2023	Jan 2024	Feb 2024	Mar 2024
Initial design and searches	✓										
Screening of duplicates and titles		✓									
Screening of abstracts			✓								
Screening of full-text articles				✓	✓						
Search references of included studies					✓						
Data extraction					✓	✓	✓				
Synthesis and risk of bias assessment								✓	✓	✓	
Abstract and manuscript drafting										✓	✓
Submission to conferences and peer-reviewed journals											✓

## Discussion

### Overview

Our proposed systematic review will be a comprehensive review that synthesizes the effects of resistance training on the health outcomes of patients after MBS. We anticipate that this study will demonstrate the benefits of resistance training on weight loss, weight maintenance, increased strength, increased bone density, maintenance of lean muscle mass, and improvement in comorbid conditions. In addition, we anticipate there will be an improvement in mental health, self-esteem, and quality of life.

The results of our review will be used to design resistance training protocols that may be prescribed to patients after MBS for optimal weight management and strength gains, as this population is at high risk of fat-free mass loss due to their very low-calorie diets and rapid weight loss. This will subsequently improve longevity in this patient population, as age-related sarcopenia, fragility, and increased risk of falls and fractures have high mortality rates [13]. Lastly, the results of this study will generate pertinent considerations for future research to continue improving the knowledge available on the benefits and application of resistance training in patients after MBS. If results from the review are suitable for pooling, this will be the largest review with quantitative analysis specific to resistance training protocols in patients after MBS.

Ultimately, the optimal protocol for resistance training is the one the patient will adhere to. Even with a clearly superior protocol, if the adherence rate is low, it will be of minimal benefit to the patient. According to a review by Bellicha et al [24], most patients do not reach the recommended levels of physical activity after bariatric surgery. A recent systematic review by Bond et al [25] demonstrated that participants who were assigned to the exercise interventions achieved only modest weight loss after 12 months, although none of the exercise interventions aligned with the 250-300 weekly minutes of moderate-intensity physical activity recommended by the American College of Sports Medicine and the 2018 physical activity guidelines for the management of obesity and prevention of weight recurrence. This study also showed that adherence rates were only reported in 2 studies [25]. The ideal protocol will look different for all patients; however, this study may provide further insight on which protocols have the highest adherence rates and what the characteristics of those protocols are. We will distinguish if there are differences in supervised versus nonsupervised sessions as well as individual versus group settings. Studies have shown that a critical component of sustained weight loss includes a sense of “reinvention” and an identity shift to a new lifestyle after successful weight loss [26,27]. Whatever the superior protocol is, it must be sustainable and habit-forming for patients who have undergone MBS.

### Limitations

This systematic review will have limitations. There will be a degree of heterogeneity among the studies selected due to the variability of the research methodologies, settings, exercise interventions, diets, and outcome measurements. There is an inherent risk of reviewer bias, which will be mitigated by the use of tools for assessing risk of bias. We will attempt to minimize the bias in this review by following strategies: (1) we will perform a sensitivity analysis to examine if overall findings are sensitive to the exclusion of a study with low quality or a risk of bias; (2) we will ensure transparency on the methods; (3) at every stage of the systematic review, we will solicit inputs from subject matter experts; and (4) we will perform a risk of bias assessment in each study using the standardized tools. There is also a risk of missing pertinent literature on our research topic. A medical librarian at the first author’s home institution was consulted and assisted in developing a broad search strategy to maximize the likelihood of identifying relevant articles. Additionally, many aspects related to resistance training are fully subjective, and many outcomes are not reflected in a continuous mathematical figure. There is a chance that any recommendations made after this study may not be statistically significant, but the goal is to perform as comprehensive a review on multiple study methodologies as possible and to report on multiple outcomes of interest that may potentially guide protocols in the future.

### Dissemination Plan

The findings from this systematic review will be disseminated through a peer-reviewed journal article and presentations at academic conferences.

### Conclusion

This study will provide a comprehensive review of the effects of resistance training in patients who have undergone MBS and will include data on outcomes regarding changes in body composition, strength, relevant health outcomes, adherence, and quality of life. This research will hopefully lead to the development of optimal resistance training practice recommendations for this patient population. This study may also shed light on a resistance training protocol that is best for adherence and long-term weight loss maintenance. With these results, we hope to provide a deeper understanding of the role of resistance training after MBS.

## Appendix

Multimedia Appendix 1 Detailed search strategy.

## Abbreviations

%EBWL: excess body weight loss
%TBWL: total body weight loss
MBS: metabolic and bariatric surgery
MD: mean difference
PECOS: Population, Experiment, Comparator, Outcome, Study Design
PI: prediction interval
PRESS: Peer Review of Electronic Search Strategies
PRISMA: Preferred Reporting Items for Systematic Reviews and Meta-Analyses

## Data Availability

The data sets generated during and/or analyzed during this study are available from the corresponding author on reasonable request.

## References

[1] Schauer PR, Bhatt DL, Kirwan JP, Wolski K, Aminian A, Brethauer SA, Navaneethan SD, Singh RP, Pothier CE, Nissen SE, Kashyap SR (2017). Bariatric surgery versus intensive medical therapy for diabetes—5-year outcomes. N Engl J Med.

[2] Aminian A, Wilson R, Al-Kurd A, Tu C, Milinovich A, Kroh M, Rosenthal RJ, Brethauer SA, Schauer PR, Kattan MW, Brown JC, Berger NA, Abraham J, Nissen SE (2022). Association of bariatric surgery with cancer risk and mortality in adults with obesity. JAMA.

[3] Verrastro O, Panunzi S, Castagneto-Gissey L, De Gaetano A, Lembo E, Capristo E, Guidone C, Angelini G, Pennestrì F, Sessa L, Vecchio FM, Riccardi L, Zocco MA, Boskoski I, Casella-Mariolo JR, Marini P, Pompili M, Casella G, Fiori E, Rubino F, Bornstein SR, Raffaelli M, Mingrone G (2023). Bariatric-metabolic surgery versus lifestyle intervention plus best medical care in non-alcoholic steatohepatitis (BRAVES): a multicentre, open-label, randomised trial. Lancet.

[4] Eisenberg D, Shikora SA, Aarts E, Aminian A, Angrisani L, Cohen RV, de Luca M, Faria SL, Goodpaster KPS, Haddad A, Himpens JM, Kow L, Kurian M, Loi K, Mahawar K, Nimeri A, O'Kane M, Papasavas PK, Ponce J, Pratt JSA, Rogers AM, Steele KE, Suter M, Kothari SN (2023). 2022 American Society of Metabolic and Bariatric Surgery (ASMBS) and International Federation for the Surgery of Obesity and Metabolic Disorders (IFSO) indications for metabolic and bariatric surgery. Obes Surg.

[5] Oppert JM, Bellicha A, van Baak MA, Battista F, Beaulieu K, Blundell JE, Carraça EV, Encantado J, Ermolao A, Pramono A, Farpour-Lambert N, Woodward E, Dicker D, Busetto L (2021). Exercise training in the management of overweight and obesity in adults: synthesis of the evidence and recommendations from the European Association for the study of obesity physical activity working group. Obes Rev.

[6] Liguori G, Roy B, Fountaine CJ, Feito Y, American College of Sports Medicine (2021). ACSM's Guidelines for Exercise Testing and Prescription, 11th Edition.

[7] Vaurs C, Diméglio C, Charras L, Anduze Y, du Rieu MC, Ritz P (2015). Determinants of changes in muscle mass after bariatric surgery. Diabetes Metab.

[8] Herring LY, Stevinson C, Davies MJ, Biddle SJ, Sutton C, Bowrey D, Carter P (2016). Changes in physical activity behaviour and physical function after bariatric surgery: a systematic review and meta-analysis. Obes Rev.

[9] Oppert JM, Bellicha A, Roda C, Bouillot JL, Torcivia A, Clement K, Poitou C, Ciangura C (2018). Resistance training and protein supplementation increase strength after bariatric surgery: a randomized controlled trial. Obesity (Silver Spring).

[10] Davidson LE, Yu W, Goodpaster BH, DeLany JP, Widen E, Lemos T, Strain GW, Pomp A, Courcoulas AP, Lin S, Janumala I, Thornton JC, Gallagher D (2018). Fat-free mass and skeletal muscle mass five years after bariatric surgery. Obesity (Silver Spring).

[11] In G, Taskin HE, Al M, Alptekin HK, Zengin K, Yumuk V, Ikitimur B (2021). Comparison of 12-week fitness protocols following bariatric surgery: aerobic exercise versus aerobic exercise and progressive resistance. Obes Surg.

[12] Ibañez J, Izquierdo M, Argüelles I, Forga L, Larrión JL, García-Unciti M, Idoate F, Gorostiaga EM (2005). Twice-weekly progressive resistance training decreases abdominal fat and improves insulin sensitivity in older men with type 2 diabetes. Diabetes Care.

[13] Westcott WL (2012). Resistance training is medicine: effects of strength training on health. Curr Sports Med Rep.

[14] Paillard T, Rolland Y, de Souto Barreto P (2015). Protective effects of physical exercise in Alzheimer's disease and Parkinson's disease: a narrative review. J Clin Neurol.

[15] FitzGerald SJ, Barlow CE, Kampert JB, Morrow JR, Jackson AW, Blair SN (2022). Muscular fitness and all-cause mortality: prospective observations. J Phys Act Health.

[16] Morales-Marroquin E, Kohl HW, Knell G, de la Cruz-Muñoz N, Messiah SE (2020). Resistance training in post-metabolic and bariatric surgery patients: a systematic review. Obes Surg.

[17] PROSPERO international prospective register of systematic reviews. National Institute for Health and Care Research.

[18] (2006). Systematic Reviews: CRD’s Guidance for Undertaking Reviews in Health Care.

[19] Bramer WM, de Jonge GB, Rethlefsen ML, Mast F, Kleijnen J (2018). A systematic approach to searching: an efficient and complete method to develop literature searches. J Med Libr Assoc.

[20] McGowan J, Sampson M, Salzwedel DM, Cogo E, Foerster V, Lefebvre C (2016). PRESS peer review of electronic search strategies: 2015 guideline statement. J Clin Epidemiol.

[21] Page MJ, McKenzie JE, Bossuyt PM, Boutron I, Hoffmann TC, Mulrow CD, Shamseer L, Tetzlaff JM, Akl EA, Brennan SE, Chou R, Glanville J, Grimshaw JM, Hróbjartsson A, Lalu MM, Li T, Loder EW, Mayo-Wilson E, McDonald S, McGuinness LA, Stewart LA, Thomas J, Tricco AC, Welch VA, Whiting P, Moher D (2021). The PRISMA 2020 statement: an updated guideline for reporting systematic reviews. BMJ.

[22] DerSimonian R, Laird N (1986). Meta-analysis in clinical trials. Control Clin Trials.

[23] Egger M, Smith GD, Schneider M, Minder C (1997). Bias in meta-analysis detected by a simple, graphical test. BMJ.

[24] Bellicha A, van Baak MA, Battista F, Beaulieu K, Blundell JE, Busetto L, Carraça EV, Dicker D, Encantado J, Ermolao A, Farpour-Lambert N, Pramono A, Woodward E, Oppert JM (2021). Effect of exercise training before and after bariatric surgery: a systematic review and meta-analysis. Obes Rev.

[25] Bond DS, Manuel KM, Wu Y, Livingston J, Papasavas PK, Baillot A, Pescatello LS (2023). Exercise for counteracting weight recurrence after bariatric surgery: a systematic review and meta-analysis of randomized controlled trials. Surg Obes Relat Dis.

[26] Epiphaniou E, Ogden J (2010). Successful weight loss maintenance and a shift in identity: from restriction to a new liberated self. J Health Psychol.

[27] Spreckley M, Seidell J, Halberstadt J (2021). Perspectives into the experience of successful, substantial long-term weight-loss maintenance: a systematic review. Int J Qual Stud Health Well-being.

