# Supplementary material for: Resistance Training in Patients After Metabolic and Bariatric Surgery: Protocol for a Systematic Review
Source: JMIR Res Protoc. 2023 Nov 23;12:e49513. doi: 10.2196/49513 (PMC10704302; doi:10.2196/49513)
Supplement: Multimedia Appendix 1 [file resprot_v12i1e49513_app1.docx]

| **Database** | **Query used** | **Limit criteria** | **Results** | **Totals** |
| --- | --- | --- | --- | --- |
| **PubMed** |  |  |  |  |
| #1 | "Bariatric Surgery"[Mesh] OR "Bariatric Surger*"[tiab] OR "Metabolic Surger*"[tiab] OR "Bariatric Surgical Procedur*"[tiab] OR "Stomach Stapling"[tiab] OR "bariatric operation*"[tiab] OR "bariatric procedur*"[tiab] OR "obesity surger*"[tiab] OR "Gastric Bypass"[Mesh] OR “gastric bypass”[tiab] OR “gastroileal bypass”[tiab] OR “roux-en-y gastric bypass”[tiab] OR “stomach bypass”[tiab] OR “gastrojejunostom*”[tiab] OR “greenville gastric bypass”[tiab] OR “roux en y gastric bypass”[tiab] OR “stomach bypass”[tiab] OR "Gastrectomy"[Mesh] OR “gastrectom*”[tiab] OR “gastric sleeve”[tiab] OR “vertical sleeve gastrectomy”[tiab] OR “sleeve gastrectom*”[tiab] OR "Biliopancreatic diversion"[mesh] OR “bilio pancreatic bypass”[tiab] OR “biliopancreatic diversion*”[tiab] OR “biliopancreatic bypass*”[tiab] | 1991 to present | 90,147 |  |
| #2 | "Resistance Training"[Mesh] OR “resistance training”[tiab] OR “strength training”[tiab] OR “Strengthening Program*”[tiab] OR “resistance exercis*”[tiab] OR “resistance-type exercis*”[tiab] OR “resistance-type training”[tiab] OR "Weight lifting"[mesh] OR “weightlifting”[tiab] | 1991 to present | 28,889 |  |
| #3 | #1 AND #2 | English, Humans |  | 61 |
|  |  |  |  |  |
| **Embase** |  |  |  |  |
| #1 | ‘Bariatric Surgery’/exp OR ‘Bariatric Surger*’:ti,ab,kw OR ‘Metabolic Surger*’:ti,ab,kw OR ‘Bariatric Surgical Procedur*’:ti,ab,kw OR ‘Stomach Stapling’:ti,ab,kw OR ‘bariatric operation*’:ti,ab,kw OR ‘bariatric procedur*’:ti,ab,kw OR ‘obesity surger*’:ti,ab,kw OR ‘Gastric Bypass’/exp OR ‘gastric bypass’:ti,ab,kw OR ‘gastroileal bypass’:ti,ab,kw OR ‘roux-en-y gastric bypass’:ti,ab,kw OR ‘stomach bypass’:ti,ab,kw OR ‘gastrojejunostom*’:ti,ab,kw OR ‘greenville gastric bypass’:ti,ab,kw OR ‘roux en y gastric bypass’:ti,ab,kw OR ‘stomach bypass’:ti,ab,kw OR ‘Gastrectomy’/exp OR ‘gastrectom*’:ti,ab,kw OR ‘gastric sleeve’:ti,ab,kw OR ‘vertical sleeve gastrectomy’:ti,ab,kw OR ‘sleeve gastrectom*’:ti,ab,kw OR ‘gastric band’/exp OR 'Bioring' OR 'Heliogast' OR 'LAP-BAND' OR 'gastric band' OR ‘Biliopancreatic diversion’/exp OR ‘bilio pancreatic bypass’:ti,ab,kw OR ‘biliopancreatic diversion*’:ti,ab,kw OR ‘biliopancreatic bypass*’:ti,ab,kw | 1991 to present | 138,186 |  |
| #2 | ‘Resistance Training’/exp OR ‘resistance training’:ti,ab,kw OR ‘strength training’:ti,ab,kw OR ‘Strengthening Program*’:ti,ab,kw OR ‘resistance exercis*’:ti,ab,kw OR ‘resistance-type exercis*’:ti,ab,kw OR ‘resistance-type training’:ti,ab,kw OR ‘Weight lifting’/exp OR ‘weightlifting’:ti,ab,kw | 1991 to present | 41,102 |  |
| #3 | #1 AND #2 AND 'human'/de | Humans |  | 208 |
|  |  |  |  |  |
| **Scopus** |  |  |  |  |
| #1 | ( TITLE-ABS-KEY ( bariatric AND surgery ) OR TITLE-ABS-KEY ( gastric AND bypass ) OR TITLE-ABS-KEY ( sleeve AND gastrectomy ) OR TITLE-ABS-KEY ( gastric AND band ) OR TITLE-ABS-KEY ( biliopancreatic AND diversion ) ) | 1991 to present | 50,871 |  |
| #2 | ( TITLE-ABS-KEY ( resistance AND training ) OR TITLE-ABS-KEY ( weight AND lifting ) ) | 1991 to present | 56,341 |  |
| #3 | #1 AND #2 | English, Humans |  | 215 |
|  |  |  |  |  |
| **CINAHL** |  |  |  |  |
| #1 | bariatric surgery OR gastric bypass OR sleeve gastrectomy OR gastric band OR biliopancreatic diversion with duodenal switch | Apply related words, apply equivalent subjects, 1991 to present | 12,585 |  |
| #2 | resistance training OR weight lifting | Apply related words, apply equivalent subjects, 1991 to present | 13,110 |  |
| #3 | #1 AND #2 | English, Humans |  | 16 |
|  |  |  |  |  |
| **Web of Science** |  |  |  |  |
|  | (((((TS=(bariatric surgery)) OR TS=(gastric bypass)) OR TS=(roux en y)) OR TS=(sleeve gastrectomy)) OR TS=(gastric band)) OR TS=(biliopancreatic diversion) | 1991 to present | 63,668 |  |
|  | (TS=(resistance training)) OR TS=(weight lifting) | 1991 to present | 49,489 |  |
|  | #1 AND #2 AND AB=("population groups" not "animal models") OR (AB=(men or women or patient or female or male or subjects or adult) NOT AB="animal models") | English, Humans |  | 184 |
|  |  |  | **GRAND TOTAL** | 684 |
